# Time course analysis of RNA stability in human placenta

**DOI:** 10.1186/1471-2199-10-21

**Published:** 2009-03-10

**Authors:** Isabelle Fajardy, Emmanuelle Moitrot, Anne Vambergue, Maryse Vandersippe-Millot, Philippe Deruelle, Jean Rousseaux

**Affiliations:** 1 Centre de Biologie Pathologie, Pôle de Biochimie et Biologie Moléculaire, CHRU de Lille, Université Lille 2, France; 2 Service de Diabétologie et d'Endocrinologie, CHRU de Lille, France; 3 Service de Gynécologie Obstétrique, CHRU de Lille, France

## Abstract

**Background:**

Evaluation of RNA quality is essential for gene expression analysis, as the presence of degraded samples may influence the interpretation of expression levels. Particularly, qRT-PCR data can be affected by RNA integrity and stability. To explore systematically how RNA quality affects qRT-PCR assay performance, a set of human placenta RNA samples was generated by two protocols handlings of fresh tissue over a progressive time course of 4 days. Protocol A consists of a direct transfer of tissue into RNA-stabilizing solution (RNAlater™) solution. Protocol B uses a dissection of placenta villosities before bio banking. We tested and compared RNA yields, total RNA integrity, mRNA integrity and stability in these two protocols according to the duration of storage.

**Results:**

A long time tissue storage had little effect on the total RNA and mRNA integrity but induced changes in the transcript levels of stress-responsive genes as TNF-alpha or COX2 after 48 h. The loss of the RNA integrity was higher in the placental tissues that underwent a dissection before RNA processing by comparison with those transferred directly into RNA later™ solution. That loss is moderate, with average RIN (RNA Integration Numbers) range values of 4.5–6.05, in comparison with values of 6.44–7.22 in samples directly transferred to RNAlater™ (protocol A). Among the house keeping genes tested, the B2M is the most stable.

**Conclusion:**

This study shows that placental samples can be stored at + 4°C up to 48 h before RNA extraction without altering RNA quality. Rapid tissue handling without dissection and using RNA-stabilizing solution (RNAlater™) is a prerequisite to obtain suitable RNA integrity and stability.

## Background

Molecular tools for tissue profiling, such as real-time quantitative RT-PCR, generally require collection of fresh frozen tissues as sources of high-quality RNA. The quality of qRT-PCR data analysis is strongly related to the integrity and stability of the mRNA extracted from the tissue which is in turn dependent on tissue sample processing.

The fragile nature of RNA and the question of RNases enriched tissues such as placenta prompted us to examine the effects of storage time conditions with regard to RNA integrity and gene expression in non fixed human term placenta.

Many parameters such as delay time, mode of tissue handling, processing protection from RNAses degradation, tissue hypoxia, might influence the quality of extracted RNA [1].

Time of delivery cannot be predicted with accuracy and it is quite difficult to take in charge fresh placenta immediately after delivery. So it is difficult to standardize the delay time from delivery to sampling. The variability of gene expression also depends on the cellular homogeneity of the tissue. Placenta is an heterogeneous tissue with a large pattern of different cells with foetal and maternal areas immerged in blood. This requires a minimum of tissue washing and dissection before sampling [2].

The mode of delivery might also be important for the quality of RNA. In fact, the duration of deliveries and the tissue hypoxia is not comparable between a placenta excised by cesarean and a placenta that follows the vaginal tray. Is has been found that the duration of labor might induce an hypoxia stress with a decrease of pH and the change of the expression of a large number of genes [3].

The quality of total RNA is evaluated by the measure of its integrity and its stability. The integrity means that the pattern of total RNA ribosomal units 28S and 18S are abundant and that we have full length mRNA. The stability means an equal distribution of stable housekeeping genes despite different and heterogeneous sample conditions. It also means a stable amount of mRNA with a short half-life time.

Our purpose was to test the influence of storage of placenta at different post partum intervals (up to 96 h every 24 h) for RNA integrity and stability, taking also in account the mode of delivery and tissues handling before banking.

## Results

### pH of tissues (Fig. 1)

**Figure 1:**
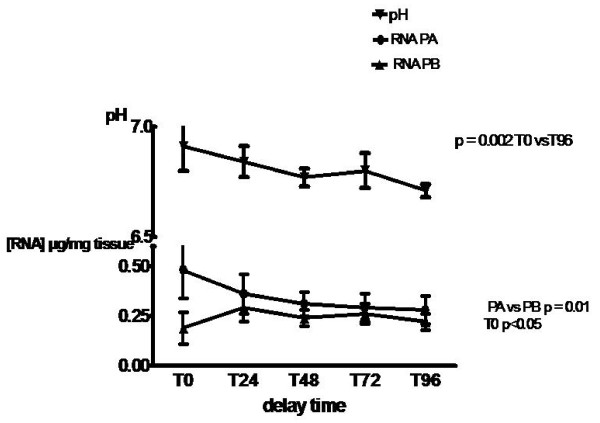
**pH and total RNA yields**. pH and total placental RNA yields after storage at +4°C from 0 to 96 h (T0 to T96). RNA samples prepared with protocol A (direct transfer to RNA later™) were compared to RNA samples prepared with protocol B (dissection before banking). Results are expressed as mean +/- SEM (each experiment in duplicate). (n = 14 for each protocol). p values were determined by ANOVA. p < 0.05 was considered to be significant. pH T0 versus T96: p = 0.002. RNA yield for protocol A versus protocol B: p = 0.01

The comparisons of pH of placenta tissue at progressive delay time showed a significant decrease at T96 (mean pH = 6.71) compared to pH = 6.9 at T0 (Kruskall Wallis test p = 0.002) (Fig. 1). At each delay time, pH values were found similar between vaginal and caesarean deliveries (data not shown).

### Evaluation of total RNA yield (Fig. 1)

We evaluated the concentration of each total RNA extract for each delay time according to handling protocol. Results were expressed as RNA yields (μg/mg of tissue). The range varies from 0.14 μg/mg to 0.57 μg/mg. The overall yield was slightly but significantly lower in protocol B (dissection of tissue before transfer) compared to protocol A (direct transfer to RNA later™) at any time (p = 0.01) (Fig. 1). We did not notice any difference of RNA concentration between vaginal and caesarean deliveries (data not shown).

### Analysis of total RNA integrity (Fig. 2)

**Figure 2:**
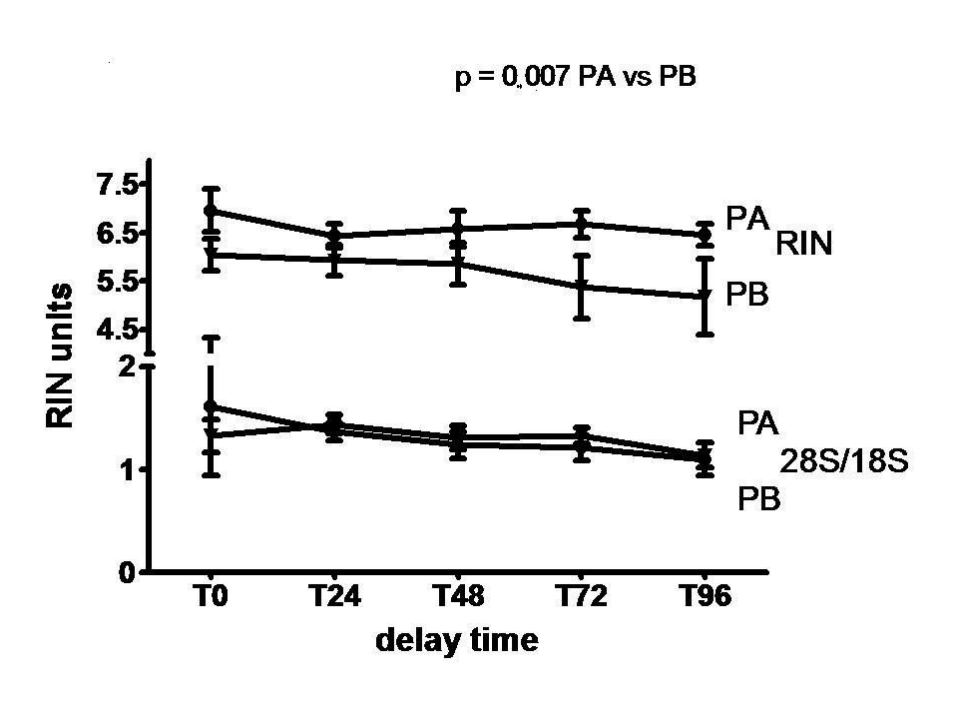
**Analysis of total RNA integrity according to delay time of storage and to handling conditions**. The integrity of total RNA in placental tissues stored at + 4°C for 0 to 96 H, was determined by Agilent Bioanalyzer assay. Results (mean +/- SEM, each experiment in duplicate) were expressed as RIN values (top of the figure), or 28S:18S ratios (bottom of the figure). RNA was prepared from placental samples transferred directly to RNA later™(protocol A), or dissected before banking (protocol B). p values were determined by ANOVA. p < 0.05 was considered to be significant. RIN protocol A versus Protocol B: p = 0.007.

Total RNA integrity was evaluated by changes of RNA Integration Numbers (RIN) and by 28S:18S values (Fig. 2).

Mean RIN values are higher in samples extracted with protocol A (range 6.44–7.22) compared to protocol B (range 4.50–6.05; p = 0.007). The two protocols differ from each other with a mean delta of 1 unit of RIN. The difference was more marked at T72 and T96 (p < 0.008). No significant decrease of RIN values with delay time was observed for the protocol A. Samples extracted with protocol B showed a significant decrease of RIN values with delay time (p = 0.05). We did not report any difference of RIN values between vaginal and caesarean deliveries (data not shown).

28S:18S ratios were similar in the two protocols with stable range (1.10–1.33) whatever handling state and delay time.

### Analysis of mRNA integrity (Fig. 3)

**Figure 3:**
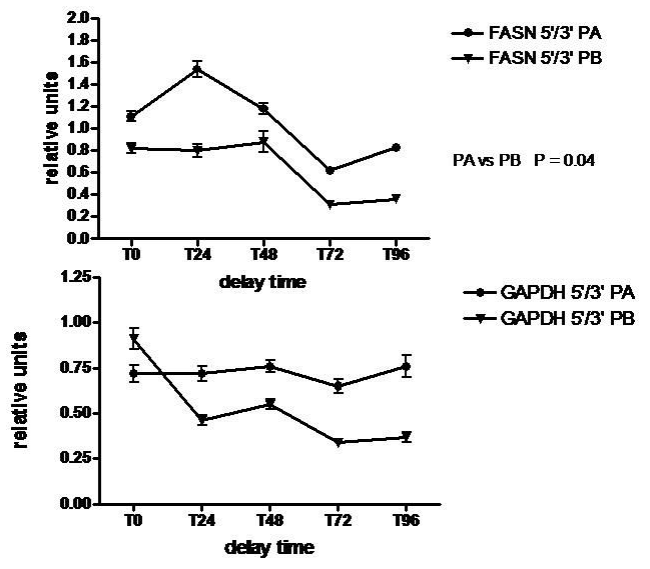
**Analysis of mRNA integrity**. Comparison of FASN (a) and GAPDH (b) mRNA 5'/3' ratios in placental samples stored at + 4°C for 0 to 96 h, as determined by qRT-PCR assays targeting sequences close to the 5' and 3' ends of the transcripts. RNA samples prepared with protocol A were compared to protocol B (n = 14 in each group). Results are expressed as mean +/- SEM. Each experiment was performed in duplicate. p values were determined by ANOVA. p < 0.05 was considered to be significant. FASN 5'/3', protocol A versus protocol B (p = 0.04), at any time of storage.

mRNA integrity was evaluated by quantification of 5' and 3' fragments of selected large house keeping genes as fatty acid synthase (FASN) (8 kb) and glyceraldéhyde-3-phosphate deshydrogenase (GAPDH) (3 kb). 5'/3' ratios around 1 value account for the integrity of the transcript. A decrease of 5' fragment is predictive of mRNA degradation [4].

FASN 5'/3' ratios varied with delay time from 1.54 to 0.62 (mean +/- SEM = 1.05 +/- 0.16) in protocol A and from 0.88 to 0.31 (mean +/- SEM = 0.63 +/- 0.18) in Protocol B (Fig. 3). The difference between the two protocols is significant (p = 0.04) assessing for a slight degradation of 5' end of FASN gene more pronounced in protocol B. These ratios decreased according to the delay time with a significant decrease at T72 compared to T0 for protocol A (p < 0.05) and protocol B (p < 0.001) respectively. Ratios were stable up to 48 h.

GAPDH 5'/3' ratios varied from 0.76 to 0.65 (mean +/- SEM 0.72 +/- 0.16) in protocol A and from 0.91 to 0.37 (mean +/- SEM = 0.53 +/- 0.11) in protocol B (Fig. 3). The difference between the two protocols is not significant. Delay time is associated with a significant decrease of 5'/3' ratio at T72 compared to T0 (p < 0.01) restricted to protocol B.

### Analysis of mRNA stability (Fig. 4 and Fig. 5)

**Figure 4:**
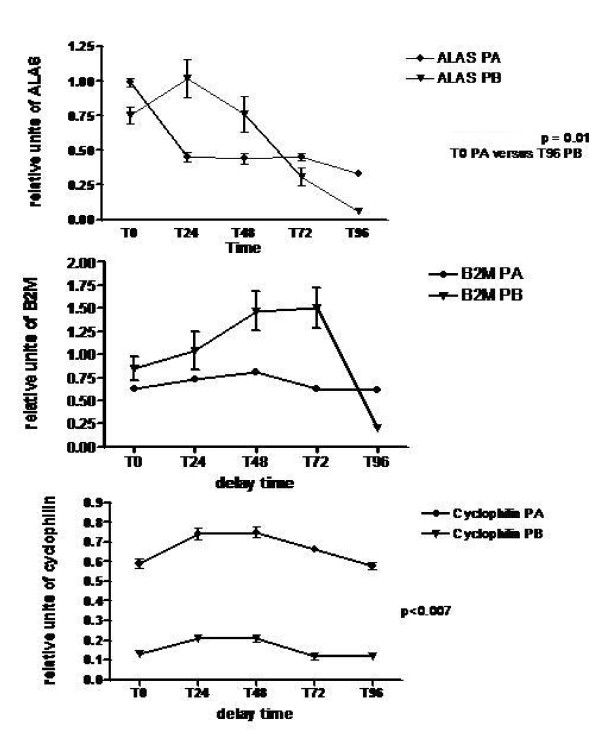
**Time course analysis of housekeeping genes**. Effect of storage time and handling conditions on housekeeping gene expression. Relative qRT-PCR amount synthetised on 1 μg of total RNA from placental tissues stored at +4°C from 0 h to 96 h according to protocol A or protocol B (n = 14 for each group). Reactions were normalised to contain equivalent amounts of total RNA. (a): ALAS (b): B2M, (c): Cyclophilin. Data are plotted as mean +/- SEM. (n = 14). p values were determined by ANOVA. p < 0.05 was considered to be significant. ALAS at T0, protocol A versus T96, protocol B: p = 0.01. Cyclophilin, protocol A versus protocol B: p < 0.007 at any time of storage.

**Figure 5:**
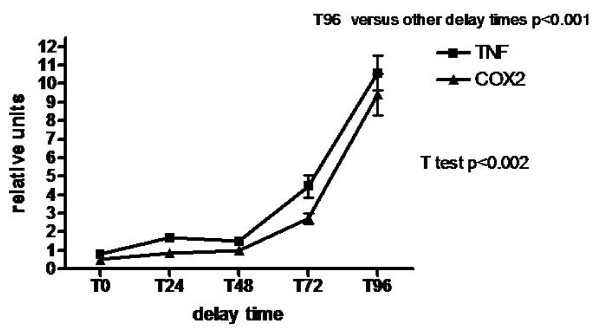
**Normalised relative abundance of TNFα and COX2 gene transcripts in placental tissues, during post partum storage at +4°C**. Geometric mean of all samples (n = 14) were normalized to the geometric mean of B2M, ALAS, and Cyclophilin, and then relative expression was calculated using the comparative Ct method. Results are expressed as mean +/- SEM. p values were determined by ANOVA. p < 0.05 was considered to be significant. T96: p < 0.002.

mRNA stability was first evaluated by the comparative expression of 3 house keeping genes: 5-aminolevulinate synthase (ALAS), β_2 _microglobulin (B2M), cyclophilin, according to tissue handling and delay time. These genes are known for their stability in placenta and therefore are routinely used for RNA normalization [5]. Fig. 4 presents the relative units in qRT-PCR obtained after correction with a calibrator.

Delay time is associated with a progressive and significant decrease of ALAS relative values in the two protocols A and B from T0 to T96 (p = 0.007 and p = 0.03 respectively). There was no difference according to tissue handling. Up to 72 h, (B2M relative values were not significantly different in the two protocols with 0.62–0.81 range in protocol A and 0.21–1.5 range in protocol B. A significant decrease of B2M expression at T96 was found for samples treated with protocol B (p < 0.01). Mean cyclophilin expression relative values were significantly different in the two protocols with lower values in protocol B: 0.66 +/- 0.08 (mean +/- SEM) in protocol A and 0.15 +/- 0.05 in protocol B (p < 0.007).

The second test for RNA stability was performed only with samples extracted with protocol A as this protocol was found to give the best stability and integrity of mRNA. Tumor necrosis factor α (TNFα) and cyclooxygenase 2 (COX2) were chosen because of the short life time of mRNA. Moreover, these two genes are immediate early response genes induced by modification of tissue like hypoxia and apoptosis [6]. Our results showed a stability of TNFα and COX2 expression up to T48 followed by a significant increase of 4 fold and 10 fold at respectively T72 and T96 (p < 0.001) (Fig. 5).

## Discussion

The limiting factor for obtaining meaningful gene expression is the quality of the initial RNA preparation. RNA purity and integrity are of foremost importance to ensure reliability and reproductibility of qRT-PCR [7]. Although the use of cell culture and laboratory animals allowed quick processing of the RNA under tightly controlled protocols, this is not always the case for human samples. It is especially true, for example, for human placenta obtained immediatly after delivery. In the studies of placental gene expression in uncomplicated pregnancies as well as in pregnancies complicated with diabetes, we were faced with the collection of placentas occuring at any hour of day and night. Thereby, time course studies of RNA expression and degradation seemed to us critically needed in order to evaluate the biostability and quality of placental RNA species, i.e how long a placental tissue may be stored without degradation of RNA. For some studies, the heterogeneity of the placenta tissue requires a previous dissection of tissue to isolate the villosities in order to study specific gene expression [8]. It was of interest to evaluate the effect on RNA yields, integrity and stability on placenta according to handling of the tissue before banking. Therefore, we evaluated the effects of post delivery delay time and tissue handling on RNA integrity and mRNA expression levels.

Reliable statistical analysis of these parameters leaded us to investigate a sufficient panel of tissues. Therefore our study was performed on 140 samples from 14 placentas, 2 protocols of preparation and 5 delay time points. Power calculations for qRT-PCR comparisons usually indicate that a sample size of at least 50 is required to detect difference. Hynd et al, reported that 13 cases by group yielded statistically differences on a range of widely disparate parameters [9].

The first parameter studied was the pH of placental tissue. pH was of interest in the light of hypoxia related to tissue injury. Hypoxia is associated with an accumulation of lactates, a lower pH and a subsequent activation of acid lysosomial RNAses [10]. There were no consistent differences in tissue pH between placentas whatever the mode of delivery. Tissue pH was found stable at + 4°C up to 72 h. A significant fall of pH was found after 96 h of storage at + 4°C. This stability has been already reported in brain tissue by others [9,11]. The overall yield of RNA was found in agreement with reports from other studies on placenta [12]. Otherwise, the yields were lower for placentas previously dissected. This suggests an activation of lysosomal RNAses by tissue disruption leading to a degradation of total RNA [7-11].

The absence of significant variation according to delay time shows that degradation of RNA seems to depend more on tissue handling than on delay time of storage. This agrees with several reports performed on various tissues [13-17].

The assessement of total RNA integrity can be done by two main methods: the standard 28S:18S ratio and the recent RIN integration method. The standard method uses electrophoresis of RNA and the evaluation of 28S and 18S bands and ratios. It is commonly accepted that intact RNA has a rRNA band ratio > 1.8 [18]. We reported very stable values in all placentas samples. The more recent method uses capillary electrophoresis and accurate integrations of peaks expressed as RIN (RNA Integration Numbers). RIN ranges from 1 to 10 with 1 being the most degraded profile and 10 the most intact [19]. In solid tissue, (6–8) RIN values are considered as valuable and reliable RNA [14]. Placenta samples dissected before extraction showed lower values than samples quickly treated with RNA later™. This significant decrease of RIN values according to handling accounts for a partial degradation of tissues by dissection. This might be explained by the activation of intracellular RNAses during tissue disruption [20]. This agrees with corresponding RNA concentrations described in Fig. 1. Several studies reported a good correlation between RIN values and qRT-PCR relative units [13,21]. They recommended then to consider a RIN > 6 for a suitable total RNA and RIN > 8 for a perfect RNA. Strand et al showed that RIN > 6 correlate with suitable expression of various genes while RIN < 6 are associated with a decrease expression of these genes [13]. Therefore, only total RNA recovered from placentas samples extracted without dissection and stored up to 96 h in RNA-later™ may be considered as reliable for qRT-PCR.

Our observations suggest that despite the 28S:18S ratio is considered as the gold standard for the measure of integrity, it lacks precision and discrimination between preserve and partially degraded RNA.

Following total RNA integrity, determination of mRNA integrity is important to assess. We analysed it by the quantification of 5' and 3' ends fragments of some gene transcripts. The fragments located towards the 5'end of the mRNA of housekeeping genes are used as indicator sequences for the degree of degradation [17,22].

FASN 5'/3' observed ratios are higher than those reported by Bauer in blood samples [22]. This might be explained by a high stability of 5' ends of genes expressed in placenta tissues [23]. FASN and GAPDH 5'/3' ratios are higher in protocol A compared to protocol B and are probably related to a partial degradation of mRNA after dissection. Expression levels of FASN and GAPDH fragments were stable up to 48 h when samples were kept at 4°C. This shows that the delay of storage has an effect on the integrity of mRNA after 48 h. This effect is higher in protocol B and fits with the decrease of total RNA integrity measured by RIN. Previous studies have observed intact total RNA in various tissues stored at + 4°C post mortem, such as human brain (up to 36 h), human bone.(up to 48 h) liver of rabbit (up to 96 h) and bovine muscle (up to 8 days) [15,17,24]. This confirms the high stability of RNA in most of tissues when stored at low temperature. The delay time has a less effect on GAPDH expression in samples treated with protocol A. In fact, we obtain very stable ratios compared to those observed with FASN. The difference of lengths of FASN and GAPDH transcripts respectively 8 kb and 3 kb might explain this difference. A very long transcript is more sensitive to partial degradation than a smaller one.

Endogenous controls, usually housekeeping genes, are measured to better normalize between tissue samples [25-28]. The choice of good controls is tissue dependant, and the same housekeeping genes suitable for a tissue are not for another [27-29]. Previous studies have compared a set of housekeeping genes in placenta by qRT-PCR [5]. B2M, ALAS and cyclophilin have been reported as stable genes in placenta and so far used in this study [5]. Our observations highlight variability of expression profiles for these 3 genes according to handling and/or delay time. B2M appears as the most stable gene no sensitive to conditions of storage or tissue handling. This agrees with a previous study showing that B2M is one of the most stable housekeeping gene in placenta [5]. ALAS mRNA expression is sensitive to storage and cyclophilin to tissue handling. This seems not to depend on the length of transcripts that are quite similar for these 3 genes. It is important to note that the length of the amplicon is over 200 bp for cyclophilin and about 100 bp for B2M and ALAS. Others reported a correlation between total RNA integrity measured by RIN and the efficiency of qRT-PCR according to the length of the PCR product [21,30]. Taken together, these results highlight that storage and handling influence the expression of standard housekeeping genes in placentas. B2M was found the most stable gene in placentas stored up to 48 h whatever the mode of preparation.

The analysis of TNFα and COX2 mRNA PCR products show a stability of expression up to 48 h and thereafter an important up regulation (4 fold and 9 fold at interval 72 h and 96 h respectively). TNFα and COX2 are involved in cellular defense and stress response. Overexpression of these genes induced by storage of rat liver at 37°C has been already reported [24]. The mechanism may be a stabilization of labile mRNA through, for example the activation of MAPK or other signaling pathways [31]. This activation of signaling pathways might be enhanced by ischemia and apoptosis of tissues during a long period of storage. Despite the little effect of delay time of storage on RNA integrity, our observation shows that it is important to take into account these variations of expression of inducible genes.

## Conclusion

This study, from the criteria of RNA yields, global RNA integrity, and RNA expression of some stable and some unstable mRNA, shows, for the first time, that it is routinely possible to obtain RNA of good quality from placentas stored intact at +4°C up to 48 h and transferred into a RNA stabilizing solution. However, one must be cautious for an extrapolation to placental expression of all the genes. Nevertheless, the delay time will be helpful for RNA preparation from this tissue, as an immediate processing is not always easy to plan. Dissection of placentas in order to obtain a tissue free of vessels and foetal membranes must be avoided or set up using a RNA-stabilizing solution.

Our results are in agreement with those from other previous studies on various human and animal tissues showing that RNA degradation is a minor problem when intact tissues are stored either at +4°C or even at room temperature before biobanking.

## Methods

### Sampling, tissue Handling

Placentas at full term (38–41 weeks of gestation), were obtained after caesarean sections or normal vaginal deliveries from 14 women with healthy babies and no complications of pregnancy. All the placentas from the patients were collected from one maternity hospital (CHRU Lille, Hôpital Jeanne de Flandre). This study is a preparative part of a PHRC approved by the Ethics Committee of LILLE (France), CPP (Comité Consultatif de Protection des Personnes dans la Recherche Biomédicale). Data were recorded anonymously. Placentas were stored at +4°C until analysis.

Biopsy placental villi (about 1 cm^3^) were removed and processed after storage of placenta at various delay times (0 h, 24 h, 48 h, 72 h, 96 h). For each placenta, tissue fragments were obtained from 4 various locations between the decidual and chorionic plates in order to limit the tissue heterogeneity [8], then submitted to 3 different treatments: (1) 150 mg of tissues were transferred immediately in a tube containing 2 ml of a commercial RNA-stabilizing solution (RNAlater™, Qiagen, SA), stored at +4°C overnight submitted to 3 different treatments: (1) 150 mg of tissues were transferred immediately in a tube containing 2 ml of a commercial RNA-stabilizing solution (RNAlater™, Qiagen, SA), stored at +4°C overnight and then moved to -80°C for long time storage (protocol A), (2) tissues were briefly washed in sterile 100 mM CaCl_2 _and PBS in order to remove blood, dissected in Petri dishes, made free of foetal membranes vessels and tissue from maternal origin, rinsed in sterile PBS solution, then adjusted to 150 mg, transferred to RNA later™ and stored at -80°C until processed (protocol B). (3) tissues were transferred to a tube containing 5 volumes of water, disrupted with a Qiagen tisssue disrupter, and the supernatant transferred to a new tube to measure the pH. All the experiments were performed on ice excepted tissue preparation for pH determination.

### RNA extraction

Each frozen sample (stored at -80°C in RNAlater™) was placed in a 13 ml tube containing 4 ml of lysing buffer solution (Qiagen, RNA easy midi Kit ™). Samples were homogenized with a Qiagen tissue disrupter using two 20–30 sec pulses, and processed for RNA isolation using the RNAeasy midi kit™ followed by an additional treatment with DNAse according to manufacturer's procedure (Qiagen). RNA concentration was evaluated by measuring the absorbance at 260 nm using the spectrophotometer Nanodrop. RNA samples extracted from placental tissues obtained from 4 various locations (see above) were pooled. Aliquots of 1 μg of pooled RNA samples were precipitated in ethanol for long storage. All tubes were RNAse free.

### RNA microchip electrophoresis

Structural RNA integrity was evaluated using a microchip electrophoresis on an AGILENT 2100 Bioanalyser (Agilent technologies) [32]. All chips (RNA6000 labchips kit) were prepared and loaded according to the manufacturer's instructions. The results were displayed as gel-like images and electrophoregrams. Total RNA degradation was evaluated using 2 criteria (1) the RNA integrity number (RIN) (2) the decrease in 28S and 18S peak areas [18] (see Additional file 1).

### cDNA synthesis

Reverse transcription was performed using the instructions for the cDNA kit (Invitrogen, ThermoScript RT-PCR system). A 20 μl reaction without reverse transcriptase was performed. A negative control without RNA was included in the reverse transcription reaction. 1 μg of total RNA was reverse transcribed at 50°C for 45 min with a mixture containing 4 μl 5 × cDNA RT buffer, 15 U Thermoscript reverse transcriptase, 1 mM dNTPs, 2.5 μM oligo-dT_20 _anchored primer, 40 U Rnase out and 5 mM DTT in a final volume of 20 μl.

### qRT-PCR analysis

Relative expression levels of RNA per sample were quantified by SYBR Green I assay on Roche Light Cycler 2700 sequence detection assay (Meylan, France). For each transcript, PCR was performed in duplicates with 10 μl reaction volumes of 1 μl of cDNA, 8 μl of mix, and 1 μl of each primer set. PCR was conducted using the following cycle parameters: 2 min at 50°C, 10 min at 95°C and 40 three steps cycles of 15 sec at 95°C, 20 sec at 50°C and 20 sec at 72°C. The assay was performed following the manufacturer's recommendations except that the reaction volume was reduced to 10 μl. A pool of cDNA from control placenta tissues prepared immediately after partum was used as a standard (in threefold serial dilutions) for quantitative correction. All cDNA samples were applied in dilution of 1:5 to obtain results within the range of the standard. Each sample was evaluated in duplicate. Analysis of transcript level was carried out using first the determination of the threshold cycle Ct for each reaction corrected by the efficiency. Then the delta Ct was calculated by subtracting the mean Ct of the calibrator from each value of Ct for each gene. The amount of target relative to a calibrator was computed by 2 ^-delta Ct^

### cDNA evaluation of integrity

cDNA integrity was investigated by comparing the qRT-PCR 5'/3' ratio for 2 selected genes: glyceraldehyde-3-phosphate dehydrogenase (GAPDH) and fatty acid synthase (FASN). These two genes were chosen because of their ubiquitous expression as a so-called house keeping (HK) genes and for their various sizes, short for GAPDH (1 kb) and large for FASN (>8 kb).

Two primer pairs generating amplification products of different sizes were spaced at 5' and 3' ends along the FASN cDNA generating fragments of 197 and 281 bp [22]. Two primers pairs for GAPDH cDNA were designed using primer3 software  that generates fragments located at 3' and 5' ends of cDNA (Table 1). This method takes advantage of the fact that the oligo-dT primed cDNA population contains complementary DNA that extends from the 3'-end to the 5'-mRNA cap structure in intact mRNA. The 5'end of the mRNA will be underrepresented in the cDNA population to a degree corresponding to the extent of degradation in the RNA preparation. The relative yields of the amplification products from 5' end to 3' end of the mRNA may be used as a relative measure of the fraction of fragmented versus intact FASN and GAPDH mRNA in the sample. All FASN and GAPDH primer binding sites were located on different exons so that avoiding contaminating genomic DNA. Results were expressed as 5'/3' ratio of relative values obtained for each gene fragment.

**Table 1:** nucleotide sequences of primers and the PCR products size

TARGET	sequences (primer 3 design) or Qiagen Geneglobe reference number	NCBI reference gene number	NCBI detected transcript number	lenght of amplicon	amplified exons*
**GAPDH (fragments)**		4141	NM_002046		
GAPDH 5'end					
GAPDH 5' Forward	5'-GAAGGTGAAGGTCGGAGT-3'			252 bp	2/3
GAPDH 5' Reverse	5'-GAAGATGGTGATGGGATTTC-3'				
GAPDH 3' end					
GAPDH 3' Forward	5'-AAACCTGCCAAATATGATGACAT-3'			228 bp	8/9
GAPDH 3' Reverse	5'-ACCCTGTTGCTGTAGCCAAA-3'				
					
**FASN (fragments)**		2194	NM_004104		
FASN 5' end					
FASN 5' Forward	5'-ATCCGCTCGTTGTACCAGTC-3'			281 bp	7
FASN 5' Reverse	5'-GATCTCAGGGTTGGGGCTAT-3'				9
FASN 3' end					
FASN 3' Forward	5'-GGTCTTGAGAGATGGCTTGC-3'			197 bp	34/35
FASN 3' Reverse	5'-TTGGCAAAGCCGTAGTTG-3'				36
					
**House keeping genes**					
ALAS1 (Aminolevulinate delta synthase 1)	QT00073122	211	NM_000688	113 pb	9/10
B2M (beta2 microglobulin)	QT00088935	567	NM_004048	98 pb	1/2
Cyclophilin A (peptidylprolyl isomerase)					
Forward	5'-TGCTGACTGTGGACAACTCGA-3'	5478	NM_203431	212 bp	4
Reverse	5'-TCATAATCATAAACTTAACTCTGCA ATCC-3'				
					
**other genes**					
TNFalpha(Tumor necrosis factor)	QT01079561	7124	NM_000594	104 bp	1/2/3
COX 2 (cyclooxygenase 2)	QT00040586	5743	NM_000963	68 bp	1/2

Detail of primers sets specified for RT-PCR analysis

Some primers are designed by Qiagen manufacturer (Gene globe Quantitect primer assay products). Others are designed using Primer 3 software.

(*) primer sets are designed to cross exon/intron boundaries in order to prevent coamplification of genomic DNA.

### mRNA evaluation of stability

Stability of mRNA was evaluated by quantification of a first set of selected HK genes almost stable: 5-aminolevulinate synthase (ALAS), β_2 _microglobulin (B2M), and cyclophilin, and a second set of 2 genes with a short half life: TNFα (37 min) and COX2 (3 h). Primers were designed by Qiagen (GENGLOBE) for ALAS and B2M or by primer3 software for cyclophilin (Table 1). TNF and COX2 relative expression were normalised by subtracting the mean geometric Ct of the 3 HK genes to the each Ct using geNorm software. Results were expressed as 2 ^-deltadeltaCt ^[33].

### Statistical analysis

To examine whether the variable qRT-PCR amounts, RNA concentrations, RIN, pH values 28S:18S ratios, were different between the groups defined by pre analytical conditions and the delay time of conservation, we used either the Kruskal-Wallis test (3 groups minimum) or the Mann-Whitney test (2 groups) which are non parametric alternative to one-way Anova.

All statistical analyses were performed using ABI PRISM software. p values less than 0.05 were considered statistically significant.

## Authors' contributions

IF designed the study, carried out the analysis and interpretation of the results and drafts the manuscript, EM contributed to the development of methodology and performed the experimental procedure, AV and PD provide placenta tissues and are physicians involved in a common clinical research project on foetal growth and gene expression in placenta, MVM assisted in the preparation of RNA. JR participated in the conception and design of the study and helped to draft the manuscript. All authors read and approved the final manuscript.

## Supplementary Material

Additional file 1 **Electrophoretic tracings of RNA according to different handling methods.** the data provived presented AGILENT graphs of two RNA samples extracted from the same tissue and submitted to Protocol A: RIN 7.2 value (a) and protocol B: RIN 4.5 value (b). Click here for file
