# Supplementary material for: Time course analysis of RNA stability in human placenta
Source: BMC Mol Biol. 2009 Mar 10;10:21. doi: 10.1186/1471-2199-10-21 (PMC2664811; doi:10.1186/1471-2199-10-21)

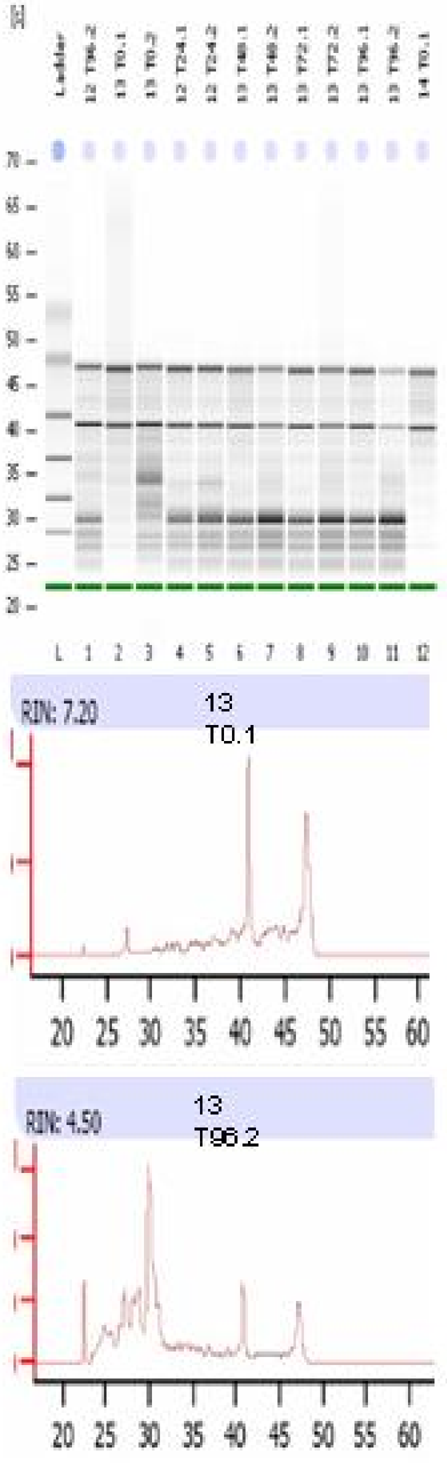

RIN: 7.20
13
T0.1
20 25 30 35 40 45 50 55 60
RIN: 4.50
13
T96.2
20 25 30 35 40 45 50 55 60

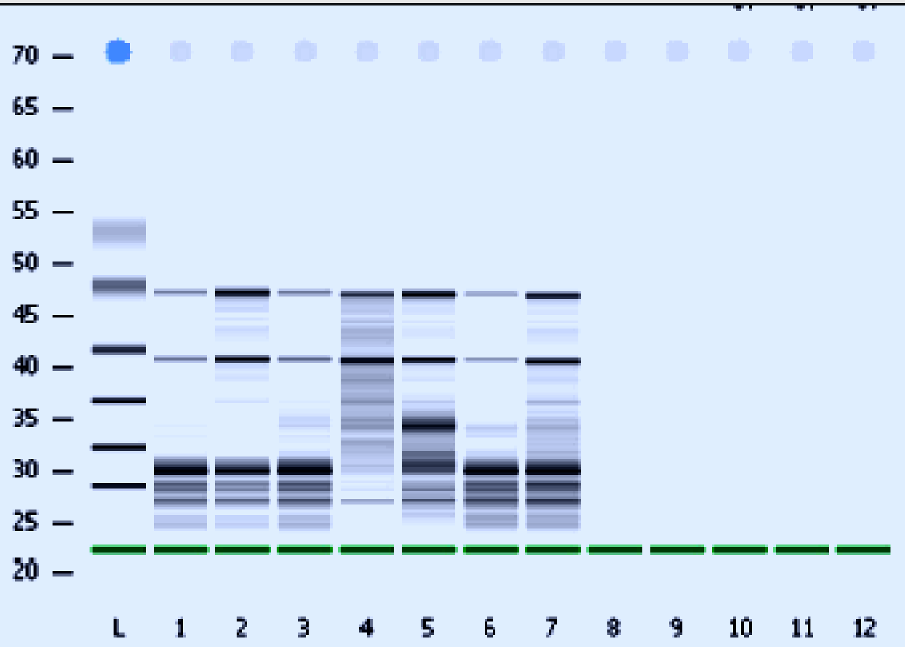


**Assay Information:**

| | |
|---|---|
| Assay Origin Path: | C:\Program Files\Agilent\2100 bioanalyzer\2100 expert\assays\RNA\Eukaryote Total RNA Nano Series II.xsy |
| Title: | Eukaryote Total RNA Nano Series II |
| Version: | 2.5 |
| Assay Comments: | Copyright © 2003-2006 Agilent Technologies |

**Chip Information:**

Chip Lot:

Reagent Kit Lot:

Chip Comments:

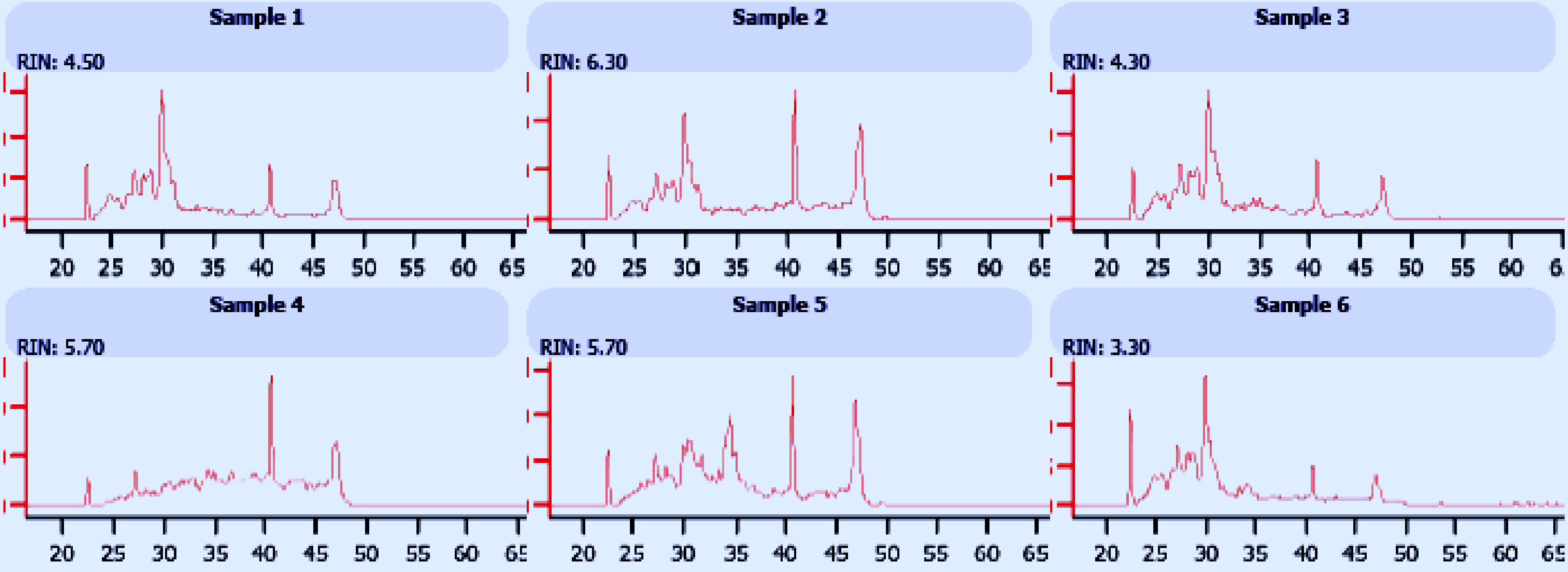

Supplement: Additional file 1 — Electrophoretic tracings of RNA according to different handling methods. the data provived presented AGILENT graphs of two RNA samples extracted from the same tissue and submitted to Protocol A: RIN 7.2 value (a) and protocol B: RIN 4.5 value (b). [file 1471-2199-10-21-S1.pdf]
